# The Apoplastic and Symplastic Antioxidant System in Onion: Response to Long-Term Salt Stress

**DOI:** 10.3390/antiox9010067

**Published:** 2020-01-12

**Authors:** Grisaly García, María José Clemente-Moreno, Pedro Díaz-Vivancos, Marina García, José Antonio Hernández

**Affiliations:** 1 Departamento de Ciencias Biológicas, Decanato de Agronomía, Universidad Centroccidental Lisandro Alvarado UCLA, Barquisimeto 3001, Estado Lara, Venezuela; grisalygarcia@ucla.edu.ve; 2 Grupo de Biotecnología de Frutales, Centro de Edafología y Biología Aplicada del Segura (CEBAS-CSIC), 30100 Murcia, Spain; esojariam2@gmail.com (M.J.C.-M.); pdv1@um.es (P.D.-V.); 3 Facultad de Ingeniería Agronómica, Universidad Técnica de Manabí. Portoviejo, Manabí 130105, Ecuador; marinabotanica@gmail.com; 4 Instituto de Botánica Agrícola, Facultad de Agronomía, Universidad Central de Venezuela, Av. 19 de abril, Maracay 1050, Estado Aragua, Venezuela

**Keywords:** *Allium cepa*, antioxidant defenses, apoplast, extracellular antioxidants, oxidative damage, salinity, symplast

## Abstract

The response of apoplastic antioxidant systems in root and leaf tissues from two onion genotypes (‘Texas 502’, salt-sensitive and ‘Granex 429’, salt-resistant) in response to salinity was studied. Electrolyte leakage data indicated the membrane integrity impairing by the effect of salts, especially in ‘Texas 502’. We detected superoxide dismutase (SOD) and peroxidase (POX) activity in the root and leaf apoplastic fractions from onion plants. Salinity increased SOD activity in the root symplast of ‘Texas 502’ and in ‘Granex 429’ leaves. In contrast, salinity reduced SOD activity in the leaf and root apoplastic fractions from ‘Texas 502’. In ‘Granex 429’, salt-stress increased leaf apoplastic POX activity and symplastic catalase (CAT) activity of both organs, but a decline in root apoplastic POX from ‘Texas 502’ took place. Salt-stress increased monodehydroascorbate reductase (MDHAR) in root and leaf symplast and in root glutathione reductase GR, mainly in ‘Granex 429’, but only in this genotype, leaf dehydroascorbate reductase (DHAR) activity increased. In contrast, a decline in leaf GR was produced only in ‘Texas 502’. Salinity increased leaf ASC levels, and no accumulation of dehydroascorbate (DHA) was observed in roots in both cases. These responses increased the redox state of ascorbate, especially in roots. In contrast, salinity declined reduced glutathione (GSH), but oxidised glutathione (GSSG) was accumulated in leaves, decreasing the redox state of glutathione. Salinity slightly increased root GSH concentration in the salt-tolerant genotype and was unchanged in the salt-sensitive genotype, but no accumulation of GSSG was produced, favoring the rise and/or maintenance of the redox state of the glutathione. These results suggest that the lower sensitivity to salt in ‘Granex 429’ could be related to a better performance of the antioxidant machinery under salinity conditions.

## 1. Introduction

Salinity is one of the main environmental factors that limit the productivity of crop plants [1]. Salts cause alterations in key physiological processes, due to the salt-induced osmotic stress and the specific ionic effect, which in turn result in water deficit, ionic toxicity and plant nutritional imbalances [2,3]. In addition, salt stress could generate a metabolic imbalance leading to the overproduction of reactive oxygen species (ROS), such as singlet oxygen (^1^O_2_), superoxide ion (O_2_•^−^), hydrogen peroxide (H_2_O_2_) and hydroxyl radicals (**•**OH) [4]. The increased production of ROS can alter the cellular homeostasis and normal metabolism through the oxidative damage to essential macromolecules [5,6]. 

It has been demonstrated that salt-induced oxidative stress can damage the membrane lipids, affecting the structure and properties of the biological membranes [7,8]. Loss of plasma membrane integrity increases the release of ions and solutes from inside the cells, a process known as electrolyte leakage [5,9]. Saline stress also promotes ROS proliferation in the apoplast space due to metabolic alterations that affect the transfer of electrons in the membrane-cell wall interface [10].

Plants have evolved a complex antioxidative defense system to mitigate and repair the damage caused by ROS, which includes enzymatic and non-enzymatic components capable of controlling the overproduction of ROS, thus reducing the oxidative damage risk [2,11]. The main antioxidant enzymes are superoxide dismutase (SOD), peroxidase (POX), catalase (CAT), and those related to the ascorbate-glutathione (ASC-GSH) cycle, while ascorbate and glutathione are among the most important non-enzymatic antioxidants [12,13,14]. Numerous evidences have shown that the efficiency in the activity of the antioxidant system under salinity conditions can play a crucial role in preventing oxidative damage, thus increasing tolerance to this stress factor [11,15]. 

The apoplastic space, includes the outer space of the plasma membrane, the cell wall, the intercellular spaces and the apoplastic fluid [16]. In a previous paper, we observed that the 2D separation of apoplastic fluid from peach leaves revealed that the majority of the polypeptides in the apoplastic fluid had low isoelectric points, in the range of pI 4–6 [17]. The apoplast also play a role in the signaling events. In that sense, the ROS produced in the apoplast are involved in local defense responses but also in cell signaling processes [18].

Onion (*Allium cepa* L.) is a salt sensitive species [1]. In Venezuela, the valley of Quibor is the main area where it is grown although salinity is a limiting factor for its production [19,20]. Previous studies have reported intraspecific differences in the response of this species to salts [21], but little is known about the causes of this differential behavior. The effect of salt stress on the antioxidant system of onion has been barely studied. El-baky et al. (2003) compared to the degree of lipid peroxidation, CAT, SOD and POX enzymes behaviour and glutathione content in leaves of three onion varieties under saline conditions and they found that the most tolerant cultivar showed an increased antioxidant capacity [9]. In this context, the objective of the present study was to evaluate the effect of salinity on some oxidative damage parameters such as electrolyte leakage and lipid peroxidation in onion root and foliar tissues. Then, we studied how the apoplastic and symplastic antioxidant systems respond to a long-term salt challenge. To achieve this goal, the antioxidant system in roots and leaves was studied in a salt-tolerant (Granex 429) and salt-sensitive (Texas 502) onion varieties. Specifically, the levels of SOD, POX, CAT, and the ascorbate-glutathione (ASC-GSH) cycle enzymes were recorded in the symplastic and apoplastic fractions of both organs in the these onion genotypes differing in salt-tolerance, in order to determine their possible role in the differential sensitivity to salts of these genotypes.

## 2. Materials and Methods

### 2.1. Plant Material 

The onion genotypes ‘Texas 502’ and ‘Granex 429’ (Seminis Vegetable Seeds, St. Louis, MO USA), with different sensitivity to salinity [21] were used. The seeds were germinated in vermiculite, kept in dark conditions, at 25 °C for 5 days. Subsequently, the seedlings were transferred to a growth chamber adjusted to a 10-h photoperiod, temperature 25/20 °C day/night, relative humidity 60–80% and photosynthetically active radiation from 400 µmol m^−2^ s^−1^.

### 2.2. Treatments and Experimental Design

Twenty days after sowing, 128 plants were selected, 64 of each genotype, which were transplanted to 10 L containers (32 plants per container) containing Hoagland nutrient solution (diluted to 50% of its standard concentration) and provided of permanent aeration. Forty days after transplanting, saline treatment was applied to 32 plants of each genotype, incorporating a mixture of three salts into the basic nutrient solution (CaSO_4_: MgSO_4_: NaCl ratio 1:2.5:3 CE 6 dS m^−1^). These salts are predominant both in the soil and in the water used for irrigation in the horticultural zone of Lara State—Venezuela [19]. The saline treatment was prolonged for 20 days and in parallel a control group was maintained applying the nutritional solution with no salts added.

A completely randomized design was used in a 2 × 2 factorial treatment arrangement: two genotypes and two types of irrigation solution (one saline and one non-saline solution), for a total of four treatments and four repetitions with eight plants per repetition.

### 2.3. Sample Selection

Sampling was carried out at the end of the salt stress period, selecting the middle portion of the root system and the middle third of the leaf blade of the third sheet expanded downward; the plant material was immediately preserved in liquid nitrogen and then kept at −80 °C until processing.

### 2.4. Histochemical ROS Detection

Histochemical H_2_O_2_ and O_2_•^−^ staining was carried out according to Hernández et al. (2001) [10], with some modification. Briefly, the histochemical detection of H_2_O_2_ in onions leaves and roots was performed using an endogenous, peroxidase-dependent in situ histochemical staining, in which tissue samples were infiltrated with 0.05 mg mL^−1^ of 3,3′-diaminobenzidine (DAB) in 50 mM Tris-acetate buffer (pH 5.0) and incubated at 25 °C, in the dark, for 2 h. Controls were performed in the presence of 10 mM ascorbic acid.

The histochemical detection of O_2_•^−^ was performed also by infiltrating onions leaves and roots tissues directly with 0.05 mg mL^−1^ nitroblue tetrazolium (NBT) in 25 mM K-Hepes buffer (pH 7.6) and incubating at 25 °C in the dark for 4 h. Controls were performed in the presence of 10 mM MnCl_2_ (O_2_•^−^ removing reagent). In both cases, samples tissues were cleared in 50% (v/v) ethanol and photographed directly using an Olympus SZX PT stereomicroscope (Olympus, Hamburg, Germany) [10].

### 2.5. Oxidative Stress Parameters

#### 2.5.1. Electrolyte Leakage Measurement

The oxidative damage was evaluated by quantifying the electrolyte leakage (EL) [22] in the fresh root and leaf tissue. Briefly, sections of approximately 1.0 g were placed in a tube with a known volume of deionized water at 25 °C for 3 h, after which the electrical conductivity (EC) was measured. Subsequently, the container with the plant material was placed in a stove at 90 °C for 2 h, and after it reached room temperature the EC was determined again. Both readings were used to calculate electrolyte leakage using the following relationship [22]:

%EL= (EC1/EC2) × 100

where, in the case of EC1, the electrical conductivity is at 25 °C, and for EC2, the electrical conductivity is at 90 °C.

#### 2.5.2. Lipid Peroxidation

The extent of lipid peroxidation was estimated by determining the concentration of thiobarbituric acid-reactive substances (TBARS) [23].

### 2.6. Apoplast Extraction

The apoplastic fraction was isolated by vacuum-infiltration in the presence of 50 mM Tris-acetate buffer pH 6.0 (infiltration buffer). Briefly, root or leaf samples (5 g), previously washed with cold deionized water, were cut into pieces (1–2 cm^2^) and washed with the infiltration buffer. Subsequently, the root or leaf pieces were infiltrated for 3 min, at 1.0 KPa and 4 °C, with the infiltration buffer containing 2 mM CaCl_2_ and 0.2 M KCl as previously described [10]. The root or leaf material were carefully externally dried and centrifuged at 1000× *g* for 5 min at 4 °C in a 25 mL-syringe barrel placed in a centrifuge tube. The liquid recovered from the bottom of the centrifuge tube, corresponding to the apoplastic fraction plus infiltration buffer, was concentrated tenfold using concentrator filters (Centricon YM-10 Amicon, Millipore, Billerica, MA, USA) and centrifuged at 3900 g for 30 min at 4 °C to obtain approximate a volume of 1 mL. The apoplastic fraction was pre-purified by chromatography in a Sephadex G-25 NAP column (GE Healthcare, Chicago, IL, USA) equilibrated with the infiltration buffer, in order to eliminate KCl. Finally, the fractions were again concentrated about 2-fold using the same procedure mentioned above until reaching a volume of approximately 600–800 μL, which was preserved at −80 °C until its use.

### 2.7. Leaf Enzyme Extraction and Enzymatic Analyses

The symplastic fraction consisted in the remaining tissue after obtaining the apoplastic fraction, that was homogenized with a 50 mM Tris-acetate buffer pH 6.0, containing 0.1 mM EDTA, 3 mM cysteine and 0.2% Triton X-100 (v/v). The macerated tissue was centrifuged at 10,000 g for 15 min and the supernatant was pre-purified by chromatography on Sephadex G-25 NAP columns (GE Healthcare, Chicago, IL, USA). The collected fraction was preserved at −80 °C until use.

The determination of SOD, peroxidase, catalase, and the ASC-GSH cycle enzymes was carried out following protocols routinely used in our laboratory [10,24]. The SOD isoenzymes identification was carried out in the apoplastic and symplastic fractions of the root and leaf tissues. The electrophoretic separation was performed at 4 °C in 10% non-denaturing polyacrylamide gels, in a continuous system, using a vertical electrophoresis chamber Miniprotean II dual (Bio Rad, CA, USA). Electrophoresis was started at 50 V for 30 min and then at 120 V for 90 min. SOD isozymes were localized by the photochemical method [25]. Isoenzyme identification was performed by selective inhibition with 2 mM KCN or 5 mM H_2_O_2_ as specific SOD inhibitors [26].

Contamination by cytoplasmic constituents was assessed by measuring the levels of glucose-6-phosphate dehydrogenase (G6PDH) [27]. G6PDH is a cytoplasmic enzyme used as a specific marker for any plasma membrane damage that may occur during apoplast extraction by the vacuum infiltration method [28].

### 2.8. Ascorbate and Glutathione Measurements

The ascorbate content was determined by the reduction of Fe^3+^ to Fe^2+^ by reduced ascorbate (ASC) in an acidic solution [29]. Then, Fe^2+^ ions form a complex with bipyridyl, giving a pink colour that absorbs at 525 nm. A standard curve for ASC in the range 0–500 µM was used to determine ascorbate levels in onion samples.

Reduced glutathione (GSH) and GSSG were assayed recording the reduction of 5,5′-dithiobis-(2-nitrobenzoic) acid (DTNB) to 2-nitrobenzoic acid (TNB) by GSH [30]. The reaction rate was monitored by measuring the change in absorbance at 412 nm for 1 min. A standard curve was developed based on GSH in the range 0–100 µm to determine glutathione contents in onion samples.

### 2.9. Statistical Analysis

The data were analysed by one-way ANOVA using the SPSS 26.0 software (SPSS Inc., 2002, Chicago, IL, USA) software. Treatment means were separated with Duncan’s multiple range test (*p* ≤ 0.05).

## 3. Results

### 3.1. Effect of Salt Stress on Oxidative Stress Parameters

**Figure 1:**
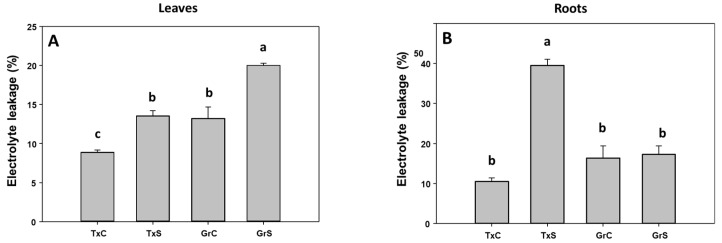
Electrolyte leakage (in %) from foliar (**A**) and root (**B**) tissues of two onion genotypes subjected to saline stress for 20 days. Different letters (a,b) indicate significant statistical difference according to Duncan’s Test (*p* < 0.05). Data represent the mean ± SE of at least four different samples. TxC (‘Texas 502’ control); TxS (‘Texas 502’, salt stressed); GrC (‘Granex 429’ control); GrS (‘Granex 429’, salt stressed).

In the two genotypes, salinity caused a significant increase in the percentage of electrolyte leakage (EL) in leaves. This raise was similar in both onion genotypes (about 65% of increase) (Figure 1A). In roots only in the salt-sensitive genotype EL strongly increased by the effect of the stress treatment, showing an enhancement of 3.7-fold compared to control values (Figure 1B). However, the effect of salt stress on the lipid peroxidation parameter was not statistically significant neither in leaves nor in roots (data not shown).

**Figure 2:**
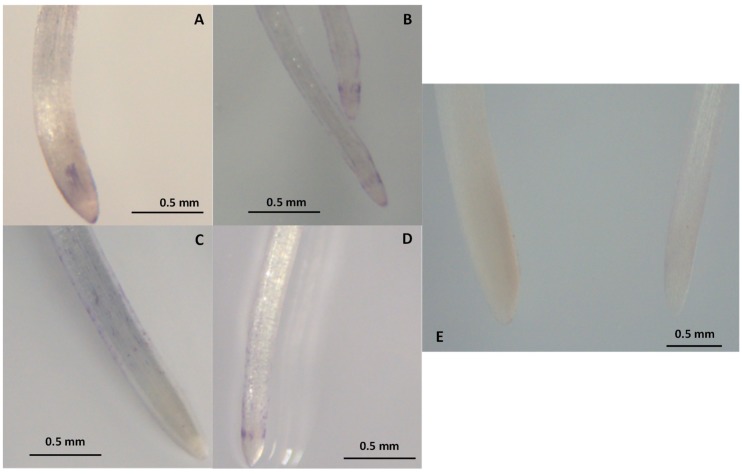
Effect of salt stress on superoxide accumulation in roots from onion plants, detected by histochemical staining with NBT. (**A**) Control ‘Texas 502’; (**B**)Salt-treated plants ‘Texas 502’; (**C**) Control ‘Granex 429’; (**D**) Salt-treated plants ‘Granex 429’; (**E**) Roots from salt-treated plants (‘Granex 429’) stained in the presence of 10 mM MnCl_2_.

**Figure 3:**
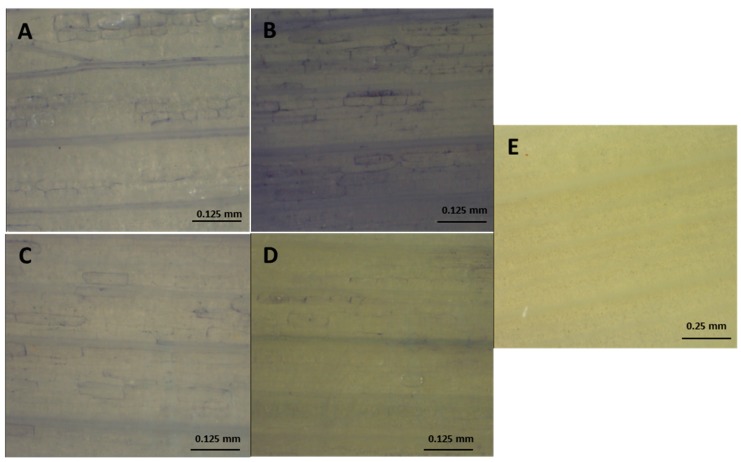
Effect of salt stress on superoxide accumulation in leaves from onion plants, detected by histochemical staining with NBT. (**A**) Control ‘Texas 502’; (**B**) Salt-treated plants ‘Texas 502’; (**C**) Detail of the previous picture (**D**) Control ‘Granex 429‘; (**E**) Salt-treated plants ‘Granex 429‘; (**F**) Leaves from salt-treated plant stained in the presence of 10 mM MnCl_2_.

The rise in electrolyte leakage was not parallel to ROS accumulation. The staining with NBT revealed an accumulation of O_2_•^−^ in the leaves and, to a lesser extent, in the roots (Figure 2 and Figure 3). In that regards, the NBT-staining was quite similar in ‘Texas 502’ roots in the absence or in the presence of NaCl. However, in ‘Granex 429’ control roots the NBT-staining was mainly located in the epidermis, while in the presence of salt stress the O_2_•^−^ accumulation seemed to be located in the root apex (Figure 2). In contrast, the NBT staining was more evident in leaves from onion plants subjected to salt stress, especially in ‘Texas 502’ leaves, that showed a higher O_2_•^−^ accumulation in the cell walls. The incubation of leaves and roots in the presence of 10 mM MnCl_2_ prevented the O_2_•^−^ staining (Figure 2E, Figure 3E.), indicating the specificity of the NBT staining. In this sense, MnCl_2_ is a highly effective dismutating catalyst agent of O_2_•^−^ [10]. However, no evident H_2_O_2_ accumulation (DAB staining) was observer in either leaves or roots (data not shown).

### 3.2. Antioxidant Enzymatic Activities

We can consider that the contamination of the leaf and the root apoplastic fractions by cytoplasmic constituents was very low. By using the enzyme G6PDH we observed percentages of contamination in the range 0.63% and 1.31% in the leaf apoplastic fraction from ‘Texas 502’, in the absence or in the presence of salt-stress, respectively. In ‘Granex 429’ the contamination of the apoplastic fractions in leaves reached 0.43% and 1.02% in the absence or in the presence of salt-stress, respectively. The cytosolic contamination in the apoplastic root fractions was lower than in leaves. In that sense, the percentages of contamination were 0.42% and 0.39% in roots from ‘Texas 502’, and 0.40% and 0.32% in roots from ‘Granex 429’ in the absence or the presence of salinity, respectively. Accordingly, the enzymatic activity data presented in the apoplastic fractions were corrected by the percentage of contamination caused by the cytosolic constituent, recorded by G6PDH activity. As expected, due to the low cytosolic contamination observed, the activities of catalase, DHAR, or GR were absent in the apoplastic fraction.

**Figure 4:**
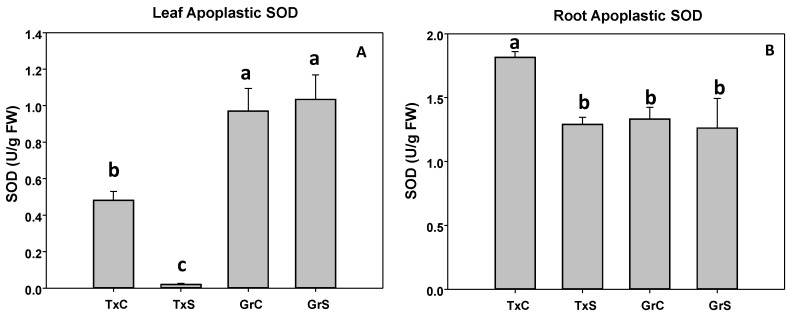
Effect of salinity in the SOD activity in the leaf (**A**) and root (**B**) apoplast in two onion genotypes differing in salinity sensibility. Different letters indicate significant statistical difference between treatments according to Duncan’s Test (*p* < 0.05). Data represent the mean ± SE of at least four different samples. TxC (‘Texas 502’ control); TxS (‘Texas 502’, salt stressed); GrC (‘Granex 429’ control); GrS (‘Granex 429’, salt stressed).

In control plants we observed that the apoplastic SOD activity represented about 0,37% of the total SOD activity in leaves and 1.53% in roots in the cultivar ‘Texas 502’, whereas in the cultivar ‘Granex 429’, apoplastic SOD represented about 0.93% and 1.13% in leaves and roots, respectively. In ‘Texas 502’ the salinity caused a significant reduction in the apoplastic SOD activity in both plant organs, especially in leaves. However, in ‘Granex 429’ no differences in SOD activity occurred in both apoplastic fractions by the effect of salinity (Figure 4A,B).

**Figure 5:**
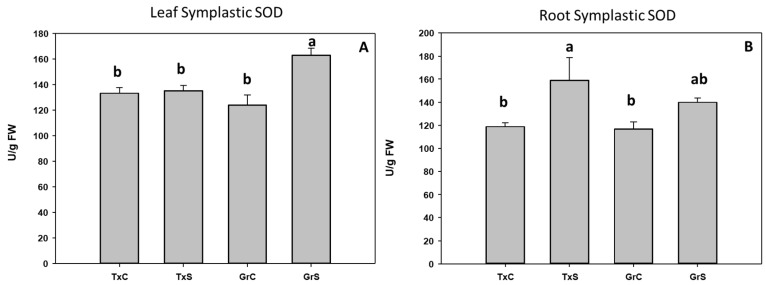
Effect of salinity in the SOD activity in the leaf (**A**) and root (**B**) symplast in two onion genotypes differing in salinity sensibility. Different letters (a,b) indicate significant statistical difference between treatments according to Duncan’s Test (*p* < 0.05). Data represent the mean ± SE of at least four different samples. TxC (‘Texas 502’ control); TxS (‘Texas 502’, salt stressed); GrC (‘Granex 429’ control); GrS (‘Granex 429’, salt stressed).

Under control conditions, both onion genotypes have similar SOD activity values in the leaf and root symplast. However, under salt stress condition a 31% increase occurred in the symplast from ‘Granex 429’ leaves compared with its control, while no changes were observed in the symplast from ‘Texas 502’ leaves. Regarding the root symplast, only in ‘Texas 502’ leaves salinity produced a significant rise of SOD activity (Figure 5).

**Figure 6:**
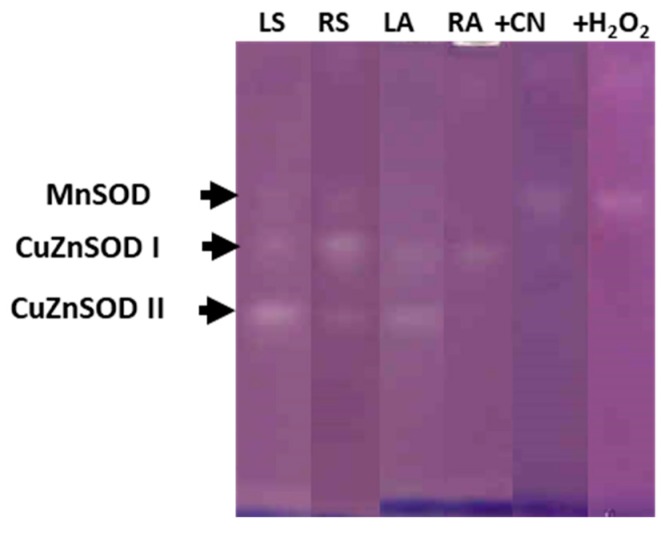
SOD isoenzyme identification after native 10% PAGE in symplast and apoplastic fractions from leaf and roots of onion ‘Texas 502’ plants. LS: Leaf symplast; RS: root symplast; LA, leaf apoplast; RA, root apoplast. + CN, incubation in the presence of 2 mM KCN. +H_2_O_2_, incubation in the presence of 5 mM H_2_O_2_. For LS and RS 50 µg of protein was used.

In both onion genotypes, the electrophoretic analysis, in presence or in the absence of the inhibitors KCN or H_2_O_2_, revealed the presence of three isoenzymes of SOD activity in the leaf and root symplasts, one Mn-SOD and two Cu,Zn-SODs, named I and II in order of increasing electrophoretic mobility (Figure 6, corresponding to ‘Texas 502 genotype’). Nevertheless, differences between organs were observed: In leaf symplast, the majority SOD isoenzyme was Cu,Zn-SOD II, whereas in root symplast, the main SOD isoenzyme was Cu,Zn-SOD I. In most plant species, Cu,Zn-SOD II has been localized in chloroplasts, whereas Cu,Zn-SOD I in mainly located in cytosol [26]. In the leaf apoplast we only observed Cu, Zn-SOD I and Cu, Zn-SOD II, this latter isoenzyme showed a lower intensity than that shown in symplast samples, and its presence in the apoplast may be motivated by a contamination caused during the purification process of the apoplast. Similar results regarding the SOD isozyme pattern in symplast and apoplastic fractions from the leaf and root of onion ‘Granex 429’ genotype were observed (Supplementary Figure S1).

**Figure 7:**
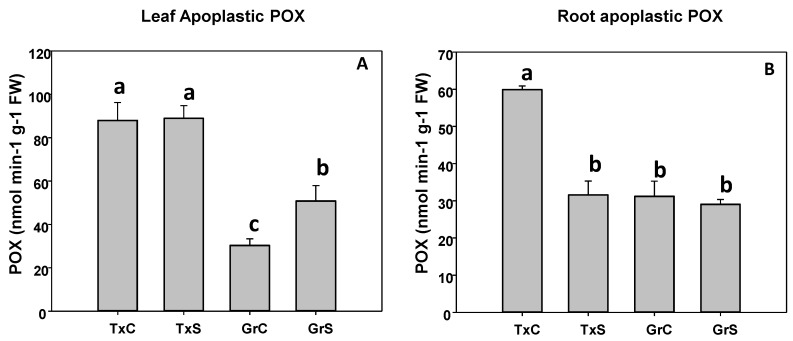
Effect of salinity in the POX activity in the leaf (**A**) and root (**B**) apoplast in two onion genotypes differing in salinity sensibility. Different letters (a–c) indicate significant statistical difference between treatments according to Duncan’s Test (*p* < 0.05). Data represent the mean ± SE of at least four different samples. TxC (Texas 502 control); TxS (Texas 502, salt stressed); GrC (Granex 429 control); GrS (Granex 429, salt stressed).

In the apoplast, POX activity was more abundant in leaf than in roots. In that regards, leaf apoplastic POX represented about 23% and 8% from the total POX activity in leaves from ‘Texas 502’ and ‘Granex 429’, respectively. In contrast, the POX activity of root apoplast represented only about 1% of the total POX activity. In addition, in both plant organs, under control conditions, apoplastic POX activity was much more abundant in ‘Texas 502’ than in ‘Granex 429’(Figure 7). The effect of salinity in apoplastic POX activity was different in the two onion genotypes. In the salt-sensitive genotype (‘Texas 502’), apoplastic POX activity did not change in leaves, but it was reduced by 40% in roots (Figure 7). In contrast, in the salt-resistant cultivar, the apoplastic POX activity increased 1.8 times in leaves and no significant changes occurred in roots (Figure 7).

**Figure 8:**
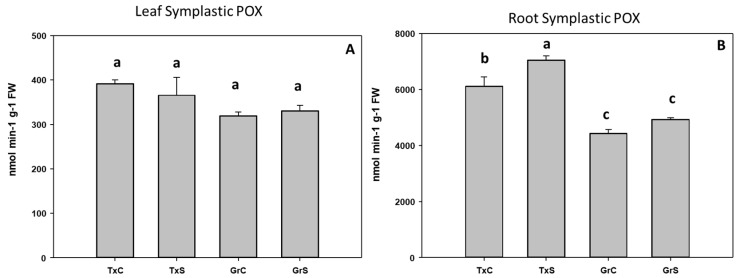
Effect of salinity in the POX activity in the leaf (**A**) and root (**B**) symplast in two onion genotypes differing in salinity sensibility. Different letters (a–c) indicate significant statistical difference between treatments according to Duncan’s Test (*p* < 0.05). Data represent the mean ± SE of at least four different samples. TxC (‘Texas 502’ control); TxS (‘Texas 502’, salt stressed); GrC (‘Granex 429’ control); GrS (‘Granex 429’, salt stressed).

In the symplast samples, POX activity was about 15–19 times higher in roots than in leaves. Salt stress only produced a significant increase in POX activity in root symplast from ‘Texas’ genotype, whereas no evident effect in leaf symplast was observed (Figure 8A,B).

**Figure 9:**
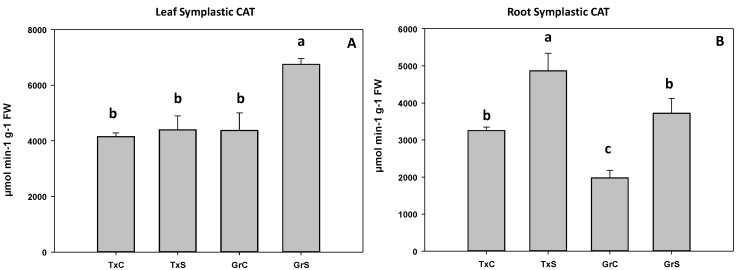
Effect of salinity in the CAT activity in the leaf (**A**) and root (**B**) symplast in two onion genotypes differing in salinity sensibility. Different letters (a–c) indicate significant statistical difference between treatments according to Duncan’s Test (*p* < 0.05). Data represent the mean ± SE of at least four different samples. TxC (‘Texas 502’ control); TxS (‘Texas 502’, salt stressed); GrC (‘Granex 429’ control); GrS (‘Granex 429’, salt stressed).

In the salt-sensitive cultivar, CAT activity did not change in leaf symplast, whereas in the salt-resistant cultivar, catalase significantly increased about 35% (Figure 9A). In the root symplast, salinity caused an increase in catalase activity in both genotypes. However, the increase was much greater in the resistant cultivar (88%) than in the sensitive cultivar (35%) (Figure 9B).

**Figure 10:**
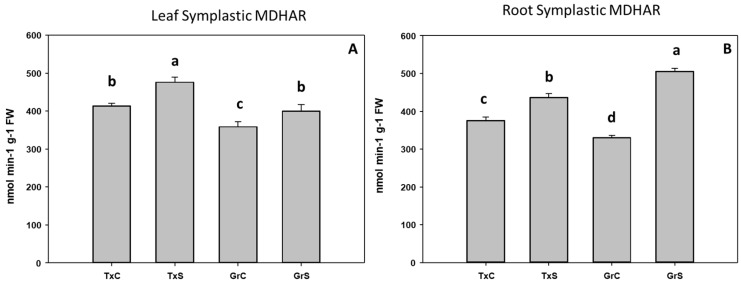
Effect of salinity in the MDHAR activity in the leaf (**A**) and root (**B**) symplast in two onion genotypes differing in salinity sensibility. Different letters (a–d) indicate significant statistical difference between treatments according to Duncan’s Test (*p* < 0.05). Data represent the mean ± SE of at least four different samples. TxC (‘Texas 502’ control); TxS (‘Texas 502’, salt stressed); GrC (‘Granex 429’ control); GrS (‘Granex 429’, salt stressed).

**Figure 11:**
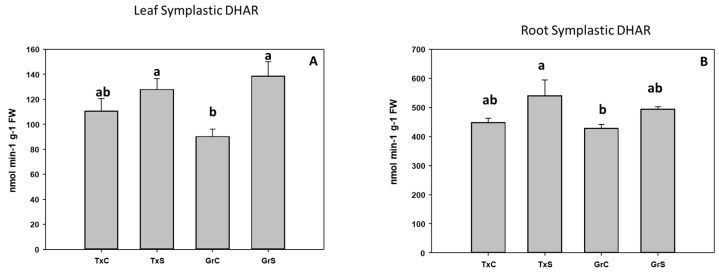
Effect of salinity in the DHAR activity in the leaf (**A**) and root (**B**) symplast in two onion genotypes differing in salinity sensibility. Different letters (a,b) indicate significant statistical difference between treatments according to Duncan’s Test (*p* < 0.05). Data represent the mean ± SE of at least four different samples. TxC (‘Texas 502’ control); TxS (‘Texas 502’, salt stressed); GrC (‘Granex 429’ control); GrS (‘Granex 429’, salt stressed).

**Figure 12:**
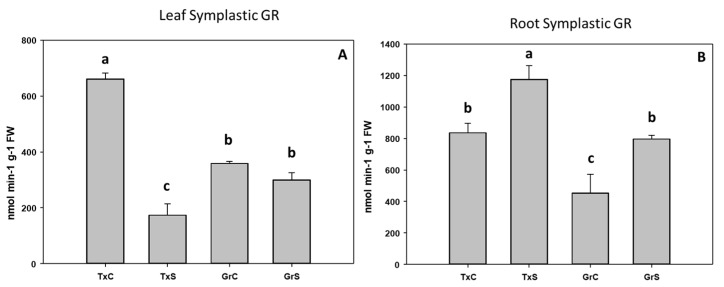
Effect of salinity in the GR activity in the leaf (**A**) and root (**B**) symplast in two onion genotypes differing in salinity sensibility. Different letters (a–c) indicate significant statistical difference between treatments according to Duncan’s Test (*p* < 0.05). Data represent the mean ± SE of at least four different samples. TxC (‘Texas 502 control); TxS (‘Texas 502’, salt stressed); GrC (‘Granex 429’ control); GrS (‘Granex 429’, salt stressed).

In addition, we also analyze the ascorbate-glutathione-recycling enzymes MDHAR, DHAR, and GR. These activities were not detected in the apoplastic fractions. Interestingly, under control conditions, the salt-sensitive genotype had higher activity values for the mentioned enzymes in the root and leaf symplast (Figure 10, Figure 11 and Figure 12). According to these data, onion roots used both ascorbate-recycling enzymes (MDHAR and DHAR) for the recycling of ascorbate. However, in leaves, the recycling of ascorbate is carried out mainly via MDHAR, using NADH as reducing power.

Under salt-stress challenge, MDHAR activity increased in the leaf symplats of both onion genotypes (Figure 10A). Under the same conditions, also an increase in MDHAR was observed in roots, being the rise much more evident in the salt-resistant genotype ‘Granex 429’ (Figure 10B). In contrast, the DHAR activity significantly increased only in the leaf symplats from the salt-resistant onion cultivar (Figure 11A). Regarding GR, a differential response was produced in roots and leaves. In leaves a decline in leaf GR of about 4-fold was produced in ‘Texas 502’, while no significant changes occurred in ‘Granex 429’ (Figure 12A). In contrast, in roots, GR undergo an increase in the presence of salt-stress, showing the salt-resistant genotype a highest increment (75%) than the salt-sensitive genotype (40%) (Figure 12B).

### 3.3. Non-Enzymatic Antioxidants

In the absence of stress, both onion genotypes contain similar levels of reduced ascorbate (ASC) in leaves and roots. Salinity stimulated the accumulation of ASC in leaves of both onion cultivars (Table 1). In addition, no accumulation of oxidized ascorbate (DHA) occurred in the presence of salt-stress. As a consequence, a slight increase in the ascorbate redox state was produced, mainly in ‘Granex 429’ (Table 1). Similarly, in roots, no significant changes in the ASC levels were observed by the effect of salt stress. However, under these conditions, the ASC contents were statistically higher in ‘Texas 502’ than in ‘Granex 429’ (Table 1). In both onion genotypes, a decline in DHA was shown under saline conditions, this effect being more evident in the salt-resistant genotype. As occurred in leaves, salinity also increased the redox state of ascorbate in roots (Table 1).

The glutathione contents were much more elevated in roots than in leaves. In the absence of salinity, ‘Texas 502’ genotype contain more reduced glutathione (GSH) than the ‘Granex 429’ genotype in leaves and in roots as well as a higher redox state of glutathione in both plant organs (Table 2). Salinity reduced the GSH levels in leaves by 50% in both onion cultivars, and increased the oxidized glutathione (GSSG) levels, resulting in a decrease in the redox state of glutathione (Table 2). In roots, no significant changes in GSH or GSSG were observed by the effect of the salinity challenge. However, in ‘Granex 429’a 38% increase in GSH was observed, producing a slight increase in the redox state of glutathione (Table 2).

## 4. Discussion

The effects of environmental stresses on the antioxidant systems of the apoplastic space have been studied by some authors, and results suggest that this compartment is very important in plant cell response to both abiotic and biotic stress conditions [10,28,31,32,33].

The effect of salinity on the antioxidant system of the leaf apoplast from pea plants was previously studied [10]. These authors reported that salinity induced an oxidative stress in the leaf apoplast, being the effects more evident in a NaCl-sensitive cultivar than in a NaCl-tolerant cultivar [10]. However, the information about the effect of salinity on the antioxidative machinery of root apoplast is very scarce. This information is very important for two main reasons. First, the root is the organ that first comes in contact with the salinity of the soil [4], and second, in plant cells subject to salt stress, initial events most likely occur externally in the apoplast-cell membrane space [10].

The results showed that salinity induced oxidative stress in leaves from both onion genotypes. Similar results have been reported by other author’s attributing these changes as a result of irregularities in the electron transport chain and accumulation of photoreducing power [5]. The effect of salt-stress in EL correlated with the degree of sensitivity to salinity between both genotypes [21]. The correlation between EL changes and salt-sensibility has been also described in other plant species such as bean, *Brasicca napus* and pea [10,22,34], confirming that EL can be an effective marker of tolerance/sensitivity to salinity in plants.

In the apoplastic space of the leaf and root from onion plants, the values found for the presence of SOD was more or less similar to data described in other plant species, including Scots pine needles and pea, oat, barley, peach and apricot leaves (0.1% to 2.5% of the total SOD activity present in the apoplast) [10,31,33]. In a recent work, it has been described that in tobacco leaves apoplastic SOD represent about 2.15% of the total SOD activity [33].

It has been suggested that the presence of POX in the apoplastic space is much more important than SOD activity. In that regards, we found a higher presence of POX in the apoplast from leaves than roots, especially in ‘Texas 502’. Under control conditions, the presence of POX in the apoplast from ‘Texas 502’ reached about 23% and 1.16% in leaf and root, respectively. In Granex 429, the presence of POX in the apoplast was somewhat lower, reaching values near 8% and 1%, respectively. These data are comparable to that obtained for the presence of POX in the apoplastic space from peach and apricot leaves [17] as well as in young branches from poplar and bitter orange plants [35]. Other authors reported values close to 10% in peach leaves and less in apricot leaves (ranging between 1.5% and 5%) for the presence of apoplastic POX activity [17]. However, in the apoplastic space from onion roots the POX activity represented about 3% of total activity [36]. These values are similar to those observed in tobacco leaves [33], where the percentage of the POX activity in the leaf apoplast reached 4.2% of the total POX activity.

Salt stress differentially affected the symplastic and the apoplastic antioxidant system in onion plants depending on their sensibility to salinity. A correlation between salt-tolerance and the response of the apoplastic antioxidant system occurred. Thus, in the salt-tolerant genotype, SOD activity was maintained in the leaf and the root apoplast fractions and increased in leaf symplast. In contrast, in the salt-sensitive cultivar only in root symplast SOD enhanced its activity, but declined in the apoplastic fractions, especially in leaves. The effect of salinity on apoplastic SOD seems to be dependent of the plant species as well as the experimental model. In pea plants, 90 mM NaCl (15 days) produced a decline in leaf apoplastic SOD of NaCl-sensible cultivar, while an increase occurred in a NaCl-tolerant cultivar [10], as occurred in the root apoplast of corn plants subjected to salinity [36]. However, in tobacco plants, leaf apoplastic SOD remained unchanged after treatment with 250 mM NaCl for 6 days [33].

This differential response in apoplastic SOD suggested that the cultivar ‘Granex 429’ has a higher capability to eliminate O_2_^.-^ radicals in the symplast and in the apoplasts from leaf and roots than the ‘Texas 502’ cultivar. This suggestion is correlated with the data obtained in the histochemical staining with NBT, showing a higher O_2_•^−^ accumulation, especially in leaf tissues. A correlation between salt-sensitivity and ROS accumulation, detected by histochemical staining with DAB (H_2_O_2_ accumulation) or NBT (O_2_•^−^ accumulation) has been described in different plants [10,37,38].

On the other hand, only in leaves from the ‘Granex 429’ genotype, salt stress produced an enhancement of the apoplastic POX in leaves; whereas in roots, salt induced a significant decrease in the apoplastic POX activity of the salt-sensitive cultivar. In tobacco plants, apoplastic POX significantly increased after six days of treatment with 250 mM NaCl. In that work, the increase in apoplastic POX (an H_2_O_2_-scavenger enzyme), along with the absence of changes in apoplastic SOD (an H_2_O_2_-generating enzyme) correlated with the absence of H_2_O_2_ accumulation in the apoplastic space [33]. Therefore, a correlation between salt-tolerance and the maintenance and/or the increase of apoplastic SOD and POX in root and leaves can be established in the salt-tolerant onion genotype. On the contrary, the decline in apoplastic POX in roots and decrease in apoplastic SOD in leaves and roots, was related with the higher sensitivity to salinity in the salt-sensitive onion genotype ‘Texas 502’.

In addition, CAT activity, that was only detected in the symplast, showed higher increases in leaves and roots from ‘Granex 429’ than in ‘Texas 502’. Similar to that described in onion plants, CAT activity was not detected in the pea leaf or tobacco apoplast [10,33]. A dramatic increase in symplastic CAT activity was also observed in the leaf symplast from tobacco plants by the effect of salt stress [33]. Increases in CAT activity have been reported in some salt-tolerant plants suggesting the involvement of photorespiration in the NaCl stress response [4]. In this sense, a correlation between CAT activity and photosynthesis has been described [39,40], since an increase in CAT reduces the photorespiratory loss of CO_2_ [41].

The different effect of salinity in the H_2_O_2_-scavenging enzymes (POX and CAT) suggests that the salt-sensitive genotype (‘Texas 502’) has a lower capacity to regulate the H_2_O_2_ that could be produced due to the salinity challenge, especially in the root apoplast.

In addition, we also analysed the enzymes MDHAR, DHAR and GR. These enzymes were not detected in the leaf or root apoplasts from onion plants. The presence of the ASC-GSH cycle enzymes in leaf apoplastic fraction is controversial. However, it has been reported the presence of all the ASC-GSH cycle enzymes in the apoplast [28,31]. Class I APX was also detected in the apoplastic fractions of poplar young branches [42], but was generally absent in the apoplastic fractions from needles of Norway spruce or of pea leaves [10,43].

Interestingly, at symplastic level onion roots seemed to use both ascorbate-recycling enzymes (MDHAR and DHAR) for the ascorbate recycling, while in leaves, the ascorbate seems to be recycled mainly via MDHAR, using NADH as reducing power. Under salt-stress challenge, the salt-tolerant cultivar increased MDHAR much more than the salt-sensitive cultivar in root. Regarding GR, a differential response was observed depending on the organ analysed, but in any case, the salt-resistant genotype experimented the best response, mainly in leaves.

It is interesting to highlight that in the absence of salt-stress, the constitutive levels of the analyzed antioxidant enzymes were in general higher in the salt-sensitive onion genotype, both in the symplastic and the apoplastic fractions. However, the salt-tolerant cultivar increased the antioxidant enzymes when grown in the presence of salinity challenge. This behavior also occurred in different cell compartments in pea plants, including the apoplastic space [10,27]. In that regards, a correlation between salt tolerance with a higher constitutive levels of some antioxidant enzymes has been described [44,45,46], whereas other authors noted that the up-regulation of the antioxidant machinery can be one of the process involved in the tolerance to salinity [10,26,27,47]. In spite of this traditional point of view, contradictory results have been also obtained, and increases in antioxidant enzymes do not always guarantee tolerance to a given stress [48].

Salinity gave rise to the same response in relation to ascorbate contents, regardless of the onion genotype studied. Salt-stress increased ASC content in leaves but not in roots in both genotypes, whereas a lower accumulation of DHA occurred in roots. These responses are correlated with the behavior of the ascorbate-regenerating enzymes. Thus, MDHAR activity increased in the leaves and roots of both onion genotypes, and in addition, only in leaves from the salt-resistant genotype was DHAR activity up-regulated.

ASC has an important role in the protection of the photosynthetic machinery at several levels: ASC have an essential role in the elimination of H_2_O_2_ produced in the Mehler reaction via ascorbate peroxidase [49]. ASC acts in the dissipation of excess light by means of the xanthofile cycle, as a co-factor for the enzyme violaxhantine de-epoxidase in the conversion of violaxhantine to zeaxhantine [50]. In addition, ASC can directly remove superoxide, hydroxyl radicals, and singlet oxygen [12]. The effect of salinity in ASC and GSH has been widely described in many plant species. In pea plants, salinity (90 mM NaCl) produced a decrease in the ASC and the GSH pools in the leaf symplast; however, this response was less evident in a salt-tolerant than in a NaCl-sensitive cultivar [10]. The effect of salt-stress decreasing the ASC or the GSH contents was also observed in soluble fraction from pea leaves, and again the effect was correlated with the degree of sensibility to salinity [27]. The effect of salinity on the ASC or GSH pool is dependent of the plant species. For example, in maize seedling, *Brassica juncea* or *Phaseolus vulgaris* plants salt stress brought about an increase in leaf ASC [5,6,51], similar to the response that we observed in onion leaves. However, in tobacco, no changes was observed in leaf apoplastic or symplastic ASC levels after treatment with 250 mM NaCl for 6 days [33].

In contrast to ASC, salinity negatively affected the levels of GSH in leaves, inducing the accumulation of GSSG. In roots, the response was somewhat different and an increase in GSH occurred, mainly in the salt-tolerant genotype, although it was not statistically significant, but produced an increase in the redox state of the glutathione content. The effect of salinity in GSH and GSSG was parallel to that of GR activity. The decrease in leaf GSH in the salt-sensitive genotype correlated to an important decrease in GR. However, salinity did not affect the GR activity in the salt-tolerant genotype, indicating that in this case salinity could alter the synthesis of GSH. In roots, the maintenance or increase of GSH contents, and the lack of accumulation of GSSG, correlated with an induction of GR activity in this organ. The salt-induced root GSH contents has been also reported in sunflower or olive plants [52,53], and this response could be linked to the mitigation of the oxidative damage provoked by the salinity in the roots, since this organ is directly exposed to the effect of salts in the soil or in the nutritive solution [4].

Taken together, the results indicate that the salt-tolerant genotype has a higher ability to scavenge O_2_•^−^ and H_2_O_2_ than the salt-sensitive genotype in symplast and apoplastic space, both in leaves and roots. In addition, regarding the differential results in root glutathione, we can also suggest that this non-enzymatic antioxidant could have a positive effect in the tolerance of onion genotype ‘Granex 429’ to long-term salinity challenge.

## 5. Conclusions

Taking together, the results obtained suggest that the lower sensitivity to salinity displayed by the ‘Granex 429’ genotype could be related to a better performance of the antioxidant machinery both in the apoplastic and the symplastic fractions. In fact, due to salt stress being first perceived by the root system, the root apoplastic antioxidant defenses were enhanced in the salt-resistant genotype compared to the salt-sensitive genotype. In addition, this effect was also observed in the apoplastic and symplastic fractions of leaves, suggesting a tight control of O_2_•^−^ and H_2_O_2_ production as well as higher ASC and GSH recycling capacity in the salt-tolerant genotype.

## Figures and Tables

**Table 1:** Effect of salinity in the content of reduced (ASC) and oxidised (DHA) ascorbate in roots and leaves from two onion genotypes.

	Genotype	Treatment	ASC	DHA	Redox State
			(nmol g^−1^ FW)		(%)
	‘Texas 502’	Control	1020.9 ^b,c^	523.4 ^a^	66.1
Leaves		Salinity	1166.1 ^a^	536.7 ^a^	68.7
	‘Granex 429’	Control	986.2 ^c^	494.2 ^a^	66.6
		Salinity	1151.3 ^a,b^	451.8 ^a^	71.8
	‘Texas 502’	Control	893.5 ^a,b^	602.3 ^a^	59.7
Roots		Salinity	1030.2 ^a^	462.3 ^b^	69.0
	‘Granex 429’	Control	961.8 ^a,b^	503.3 ^b^	65.6
		Salinity	808.1 ^b^	262.5 ^c^	75.5

Different letters (^a–c^) in each column indicates significant differences according to Duncan’s Test (*p* < 0.05); FW: Fresh weight.

**Table 2:** Effect of salinity in the content of reduced (GSH) and oxidised (GSSG) glutathione in roots and leaves from two onion genotypes.

	Genotype	Treatment	GSH	GSSG	Redox State
			(nmol g^−1^ FW)		(%)
	‘Texas 502’	Control	11.59 ^a^	2.66 ^c^	81.3
Leaves		Salino	5.84 ^b^	3.94 ^b^	59.7
	‘Granex 429’	Control	6.21 ^b^	4.42 ^b^	58.4
		Salino	3.00 ^b^	5.76 ^a^	34.2
	‘Texas 502’	Control	52.43 ^a^	9.16 ^a^	85.1
Roots		Salino	56.82 ^a^	9.20 ^a^	86.1
	‘Granex 429’	Control	34.97 ^b^	10.30 ^a^	77.2
		Salino	48.1 ^a,b^	11.55 ^a^	80.6

Different letters (^a–c^) in each column indicates significant differences according to Duncan’s Test (*p* < 0.05); FW: Fresh weight.

## Supplementary Materials

The following are available online at https://www.mdpi.com/2076-3921/9/1/67/s1, Figure S1: SOD isoenzyme identification after native 10% PAGE in symplast and apoplastic fractions from leaf and root of onion ‘Granex 429’ plants. LS: Leaf symplast; RS: root symplast; LA, leaf apoplast; RA, root apoplast. +CN, incubation in the presence of 2 mM KCN. +H_2_O_2_, incubation in the presence of 5 mM H_2_O_2_. For LS and RS 50 µg of protein was used.

## References

[1] Ashraf M., Harris J.C. (2004). Potential biochemical indicators of salinity tolerance in plants. Plant Sci..

[2] Sairam R.K., Tyagi A. (2004). Physiology and molecular biology of salinity stress tolerance in plants. Curr. Sci..

[3] Negrão S., Schmöckel S.M., Tester M. (2017). Evaluating physiological responses of plants to salinity stress. Ann. Bot..

[4] Acosta-Motos J.R., Ortuño M.F., Bernal-Vicente A., Diaz-Vivancos P., Sanchez-Blanco M.J., Hernandez J.A. (2017). Plant responses to salt stress: Adaptive mechanisms. Agronomy.

[5] AbdElgawad H., Zinta G., Hegab M.M., Pandey R., Asard H., Abuelsoud W. (2016). High salinity induces different oxidative stress and antioxidant responses in maize seedlings organs. Front. Plant Sci..

[6] Kumar M., Kumar R., Jain V., Jain S. (2018). Differential behavior of the antioxidant system in response to salinity induced oxidative stress in salt-tolerant and salt-sensitive cultivars of *Brassica juncea* L.. Biocatal. Agric. Biotechnol..

[7] Khan M.H., Panda S.K. (2008). Alterations in root lipid peroxidation and antioxidative responses in two rice cultivars under NaCl-salinity stress. Acta Physiol. Plant..

[8] Choudhury F.K., Rivero R.M., Blumwald E., Mittler R. (2017). Reactive oxygen species, abiotic stress and stress combination. Plant J..

[9] El-baky A., Hanaa H., Hussein M.M., Amal M.A. (2003). Influence of Salinity on Lipid Peroxidation, Antioxidant Enzymes and Electrophoretic Patterns of Protein and Isoenzymes in Leaves of Some Onion Cultivars. Asian J. Plant Sci..

[10] Hernandez J.A., Ferrer M.A., Jimenez A., Barcelo A.R., Sevilla F. (2001). Antioxidant systems and O_2_•^−^/H_2_O_2_ production in the apoplast of pea leaves. Its relation with salt-induced necrotic lesions in minor veins. Plant Physiol..

[11] Farooq M., Hussain M., Wakeel A., Siddique K.H.M. (2015). Salt stress in maize: Effects, resistance mechanisms, and management. A review. Agron. Sustain. Dev..

[12] Noctor G., Foyer C.H. (1998). Ascorbate and Glutathione: Keeping Active Oxygen Under Control. Annu. Rev. Plant Biol..

[13] Bartels D., Sunkar R. (2005). Drought and salt tolerance in plants. CRC. Crit. Rev. Plant Sci..

[14] Das K., Roychoudhury A. (2014). Reactive oxygen species (ROS) and response of antioxidants as ROS-scavengers during environmental stress in plants. Front. Environ. Sci..

[15] Kibria M.G., Hossain M., Murata Y., Hoque M.A. (2017). Antioxidant Defense Mechanisms of Salinity Tolerance in Rice Genotypes. Rice Sci..

[16] Guerra-Guimarães L., Pinheiro C., Chaves I., Barros D.R., Ricardo C.P. (2016). Protein dynamics in the plant extracellular space. Proteomes.

[17] Diaz-Vivancos P., Rubio M., Mesonero V., Periago P.M., Barcelo A.R., Martinez-Gomez P., Hernandez J.A. (2006). The apoplastic antioxidant system in Prunus: Response to long-term plum pox virus infection. J. Exp. Bot..

[18] Podgórska A., Burian M., Szal B. (2017). Extra-cellular but extra-ordinarily important for cells: Apoplastic reactive oxygen species metabolism. Front. Plant Sci..

[19] Villafañe R., Azpúrua M., Ruiz T., Dugarte J., Abarca O. (1999). Distribución espacial de la salinidad en los suelos de Qíbor y su relación con las limitaciones de drenaje y la calidad del agua. Bioagro.

[20] Torres D., Álvarez J., Contreras J., Henríquez M., Hernández W., Lorbes J., Mogollón J.P. (2017). Identificación De potencialidades y limitaciones de suelos agrícolas Del Estado Lara, Venezuela. Bioagro.

[21] García G., García M., Ramírez H. (2015). Comportamiento de siete cultivares de *Allium cepa* L. ante diferentes niveles de estrés salino. Bioagro.

[22] Maia J.M., de Macedo C.E.C., Voigt E.L., Freitas J.B.S., Silveira J.A.G. (2010). Antioxidative enzymatic protection in leaves of two contrasting cowpea cultivars under salinity. Biol. Plant..

[23] Hernández J.A., Almansa M.S. (2002). Short-term effects of salt stress on antioxidant systems and leaf water relations of pea leaves. Physiol. Plant..

[24] Barba-Espin G., Clemente-Moreno M.J., Alvarez S., Garcia-Legaz M.F., Hernandez J.A., Diaz-Vivancos P. (2011). Salicylic acid negatively affects the response to salt stress in pea plants. Plant Biol..

[25] Weisiger R.A., Fridovich I. (1973). Mitochondrial superoxide dismutase. Site of synthesis and intramitochondrial localization. J. Biol. Chem..

[26] Gomez J.M., Hernandez J.A., Jimenez A., del Rio L.A., Sevilla F. (1999). Differential response of antioxidative enzymes of chloroplasts and mitochondria to long-term NaCl stress of pea plants. Free Radic. Res..

[27] Hernández J.A., Jiménez A., Mullineaux P., Sevilla F. (2000). Tolerance of pea (*Pisum sativum* L.) to long-term salt stress is associated with induction of antioxidant defences. Plant Cell Environ..

[28] Vanacker H., Harbinson J., Ruisch J., Carver T.L.W., Foyer C.H. (1998). Antioxidant defences of the apoplast. Protoplasma.

[29] Law M.Y., Charles S.A., Halliwell B. (1983). Glutathione and ascorbic acid in spinach (*Spinacia oleracea*) chloroplasts. The effect of hydrogen peroxide and of Paraquat. Biochem. J..

[30] Zhang J., Kirkham M.B. (1996). Enzymatic responses of the ascorbate-glutathione cycle to drought in sorghum and sunflower plants. Plant Sci..

[31] Vanacker H., Carver T.L.W., Foyer C.H. (1998). Pathogen-induced changes in the antioxidant status of the apoplast in barley leaves. Plant Physiol..

[32] Pignocchi C., Foyer C.H. (2003). Apoplastic ascorbate metabolism and its role in the regulation of cell signalling. Curr. Opin. Plant Biol..

[33] Clemente-Moreno M.J., Gago J., Díaz-Vivancos P., Bernal A., Miedes E., Bresta P., Liakopoulos G., Fernie A.R., Hernández J.A., Flexas J. (2019). The apoplastic antioxidant system and altered cell wall dynamics influence mesophyll conductance and the rate of photosynthesis. Plant J..

[34] Ashraf M., Ali Q. (2008). Relative membrane permeability and activities of some antioxidant enzymes as the key determinants of salt tolerance in canola (*Brassica napus* L.). Environ. Exp. Bot..

[35] Bustos D., Lascano R., Villasuso A.L., Machado E., Senn M.E., Córdoba A., Taleisnik E. (2008). Reductions in maize root-tip elongation by salt and osmotic stress do not correlate with apoplastic O^2^—Levels. Ann. Bot..

[36] Córdoba-Pedregosa M.D.C., Córdoba F., Villalba J.M., González-Reyes J.A. (2003). Zonal changes in ascorbate and hydrogen peroxide contents, peroxidase, and ascorbate-related enzyme activities in onion roots. Plant Physiol..

[37] Ikbal F.E., Antonio Hernandez J., Barba-Espin G., Koussa T., Aziz A., Faize M., Diaz-Vivancos P. (2014). Enhanced salt-induced antioxidative responses involve a contribution of polyamine biosynthesis in grapevine plants. J. Plant Physiol..

[38] Cantabella D., Piqueras A., Acosta-Motos J.R., Bernal-Vicente A., Hernández J.A., Díaz-Vivancos P. (2017). Salt-tolerance mechanisms induced in Stevia rebaudiana Bertoni: Effects on mineral nutrition, antioxidative metabolism and steviol glycoside content. Plant Physiol. Biochem..

[39] Acosta-Motos J.-R., Diaz-Vivancos P., Alvarez S., Fernandez-Garcia N., Jesus Sanchez-Blanco M., Antonio Hernandez J. (2015). Physiological and biochemical mechanisms of the ornamental Eugenia myrtifolia L. plants for coping with NaCl stress and recovery. Planta.

[40] Acosta-Motos J.R., Diaz-Vivancos P., Alvarez S., Fernandez-Garcia N., Jesus Sanchez-Blanco M., Hernandez J.A. (2015). NaCl-induced physiological and biochemical adaptative mechanisms in the ornamental *Myrtus communis* L. plants. J. Plant Physiol..

[41] Brisson L.F., Zelitch I., Havir E.A. (1998). Manipulation of catalase levels produces altered photosynthesis in transgenic tobacco plants. Plant Physiol..

[42] Ros-Barceló A., Gómez-Ros L.V., Ferrer M.A., Hernández J.A. (2006). The apoplastic antioxidant enzymatic system in the wood-forming tissues of trees. Trees Struct. Funct..

[43] Polle A., Chakrabarti K., Schürmann W., Rennenberg H. (1990). Composition and properties of hydrogen peroxide decomposing systems in extracellular and total extracts from needles of Norway spruce (*Picea abies* L., karst.). Plant Physiol..

[44] Gueta-Dahan Y., Yaniv Z., Zilinskas B.A., Ben-Hayyim G. (1997). Salt and oxidative stress: Similar and specific responses and their relation to salt tolerance in citrus. Planta.

[45] López-Climent M.F., Arbona V., Pérez-Clemente R.M., Gómez-Cadenas A. (2008). Relationship between salt tolerance and photosynthetic machinery performance in citrus. Environ. Exp. Bot..

[46] Hernández J.A., Aguilar A.B., Portillo B., López-Gómez E., Beneyto J.M., García-Legaz M.F. (2003). The effect of calcium on the antioxidant enzymes from salt-treated loquat and anger plants. Funct. Plant Biol..

[47] Mittova V., Tal M., Volokita M., Guy M. (2003). Up-regulation of the leaf mitochondrial and peroxisomal antioxidative systems in response to salt-induced oxidative stress in the wild salt-tolerant tomato species *Lycopersicon pennellii*. Plant Cell Environ..

[48] Hernández J.A., Barba-Espín G., Clemente-Moreno M.J., Díaz-Vivancos P. (2016). Plant Responses to Salinity Through an Antioxidative Metabolism and Proteomic Point of View.

[49] Asada K. (2006). Production and scavenging of reactive oxygen species in chloroplasts and their functions. Plant Physiol..

[50] Demmig-Adams B., Adams W.W. (1996). The role of xanthophyll cycle carotenoids in the protection of photosynthesis. Trends Plant Sci..

[51] Taïbi K., Taïbi F., Ait Abderrahim L., Ennajah A., Belkhodja M., Mulet J.M. (2016). Effect of salt stress on growth, chlorophyll content, lipid peroxidation and antioxidant defence systems in *Phaseolus vulgaris* L.. S. Afr. J. Bot..

[52] Di Baccio D., Navari-Izzo F., Izzo R. (2004). Seawater irrigation: Antioxidant defence responses in leaves and roots of a sunflower (*Helianthus annuus* L.) ecotype. J. Plant Physiol..

[53] Aliniaeifard S., Hajilou J., Tabatabaei S.J., Sifi-Kalhor M. (2016). Effects of Ascorbic Acid and Reduced Glutathione on the Alleviation of Salinity Stress in Olive Plants. Int. J. Fruit Sci..

