# Supplementary material for: The Apoplastic and Symplastic Antioxidant System in Onion: Response to Long-Term Salt Stress
Source: Antioxidants (Basel). 2020 Jan 12;9(1):67. doi: 10.3390/antiox9010067 (PMC7022848; doi:10.3390/antiox9010067)
Supplement: Supplementary file 1 [file antioxidants-09-00067-s001.pdf]

GRANEX

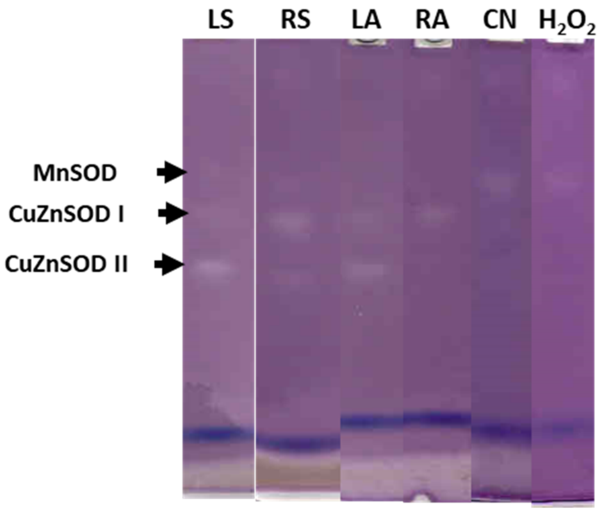

Figure S1. SOD isoenzyme identification after native 10% PAGE in symplast and apoplastic fractions from leaf and root of onion 'Granex 429' plants. LS: Leaf symplast; RS: root symplast; LA, leaf apoplast; RA, root apoplast. +CN, incubation in the presence of 2 mM KCN. +H2O2, incubation in the presence of 5 mM H2O2. For LS and RS 50 µg of protein was used.
